# Retracted publications and their citation in dental literature: A systematic review

**DOI:** 10.1002/cre2.292

**Published:** 2020-03-31

**Authors:** Antonio Rapani, Teresa Lombardi, Federico Berton, Veronica Del Lupo, Roberto Di Lenarda, Claudio Stacchi

**Affiliations:** ^1^ Department of Medical, Surgical and Health Sciences University of Trieste Trieste Italy; ^2^ Department of Health Sciences Magna Græcia University Catanzaro Italy

**Keywords:** citations, retracted publication, scientific misconduct

## Abstract

**Objectives:**

The present systematic review aimed to perform an in‐depth analysis of the different features of retracted publications in the dental field.

**Material and methods:**

This review has been recorded in the PROSPERO database (CRD42017075634). Two independent reviewers performed an electronic search (Pubmed, Retraction Watch) for retracted articles in dental literature up to December 31, 2018.

**Results:**

180 retracted papers were identified, the first published in 2001. Retractions increased by 47% in the last four‐year period (2014–2018), when compared with 2009–2013 (94 and 64 retracted publications, respectively). Author misconduct was the most common reason for retraction (65.0%), followed by honest scientific errors (12.2%) and publisher‐related issues (10.6%). The majority of retracted research was conducted in Asia (55.6%), with 49 papers written in India (27.2%). 552 researchers (89%) are listed as authors in only one retracted article, while 10 researchers (1.6%) are present in five or more retracted publications. Retracted articles were cited 530 times after retraction: the great majority of these citations (89.6%) did not consider the existence of the retraction notice and treated data from retracted articles as reliable.

**Conclusions:**

Retractions in dental literature have constantly increased in recent years, with the majority of them due to misconduct and fraud. The publication of unreliable research has many negative consequences. Studies derived from such material are designed on potentially incorrect bases, waste funds and resources, and most importantly, increase risk of incorrect treatment for patients. Citation of retracted papers represents a major issue for the scientific community.

## 1 INTRODUCTION

The publication of unreliable medical research has many negative consequences. Studies deriving from such material are designed on potentially incorrect bases, waste funds and resources, and most importantly, increase risk of incorrect therapy for patients.

The retraction of a scientific article may be decided by the journal editor or be requested directly by the author when validity of the research and its findings is seriously compromised. Reasons for retraction are usually related to authors' behaviour, either fraudulent (e.g. misconduct, plagiarism, intentional duplicate publication) or honest (e.g. methodological errors), and can sometimes be related to publisher issues (e.g. accidental duplicate publication).

Even if retractions are uncommon in medical literature, their number has significantly increased in recent years (Cokol, Ozbay, & Rodriguez‐Esteban, 2008; Steen, 2011). This could be due to tighter and more accurate control by scientific journals over acceptance and publication processes, including strict peer review and use of specific software to identify plagiarism. This tendency has also been confirmed in the field of dentistry. A recently published analysis showed that 57% of retractions in dental literature were performed after 2012 (Faggion Jr, Ware, Bakas, & Wasiak, 2018).

Unfortunately, articles are often cited even after their retraction. In both medicine (Budd, Sievert, Schultz, & Scoville, 1999; Neale, Dailey, & Abrams, 2010) and in dentistry (Faggion Jr et al., 2018) approximately 60% of retracted papers continue to be cited in literature. In these cases, however, citations may be appropriate (indicating the presence of a retraction in the manuscript and bibliographic references, and possibly discussing the questionable value of the reported data) or inappropriate (treating the findings of the retracted publication as reliable).

Therefore, the aim of the present systematic review is to perform an in‐depth analysis of the different features of retracted publications in the dental field, with particular attention paid to the presence and appropriateness of the citations received after retraction.

## 2 MATERIAL AND METHODS

### 2.1 Search strategy

The present review has been recorded in the PROSPERO database (www.crd.york.ac.uk/PROSPERO) with registration number CRD42017075634. Methods for conducting this analysis are derived from previous reviews on retracted articles in medicine and from the Cochrane Handbook for Systematic Reviews (Higgins et al., 2019).

An electronic search was conducted on Pubmed Central (PubMed, www.ncbi.nlm.nih.gov/pubmed) by two independent authors (A.R. and T.L.), selecting articles published from database inception up to the latest access on December 31, 2018. In addition, the Retraction Watch website (www.retractiondatabase.org) was carefully browsed for retracted studies in the dental and oro‐maxillofacial field. No language restriction was applied in order to limit selection bias.

### 2.2 Search

Search in the selected electronic database was performed by using the following algorithms:

Pubmed: (((retracted[Title]) OR retraction[Title]) OR withdrawn[Title]);

Retraction Watch: subject “dentistry.”

### 2.3 Selection of studies

Two blinded authors (C.S. and F.B.) independently performed eligibility assessment of the studies. Inter‐examiner reliability in the study selection process was assessed using the Cohen k‐test assuming a threshold value of 0.61 to indicate substantial agreement (Landis & Koch, 1977). Eventual conflicts were resolved by discussing each article until consensus was reached. When necessary, an attempt to contact the Editors and the Authors of the included studies was made in order to retrieve any missing information or to clarify specific items.

The following inclusion criteria were used:

1. any topic related to dental sciences, oral surgery and oral pathology;
2. all levels of scientific evidence;
3. basic science, animal and human studies.

The following exclusion criteria were applied:

1. topic unrelated to dental research;
2. use of the words “retraction” or “retracted” with a different meaning from the one considered by the present review;
3. duplicate papers.

### 2.4 Data extraction

Data were independently extracted in form of variables from the selected studies by two authors (V.D.L. and C.S.). The following items were extracted by using predefined forms:

1. authors demographics (name, nationality, affiliation);
2. journal information (journal name, impact factor [IF] in the year of publication of the retracted article, according to ISI Web of Knowledge, Thomson Reuters, Journal Citations Reports);
3. year of publication;
4. study characteristics (study design, dental subspecialty);
5. year of retraction;
6. reasons for retraction;
7. retraction characteristics (article and retraction notice availability, presence of watermark identifying the article as retracted);
8. total number of citations received by the retracted article, according to Scopus database (www.scopus.com). All articles citing a retracted paper were downloaded in full text and checked for the appropriateness of the citation. Papers citing a retracted article in the year of publication of the retraction notice and in the subsequent year were excluded from the present analysis in order to eliminate cases of citation by Authors potentially unaware of the cited document's retraction.

### 2.5 Statistical analysis

An independent examiner (R.D.L.) analysed all datasets with statistical software (Statistical Package for Social Sciences v.15, SPSS Inc., Chicago, IL). Descriptive statistics presented parametric continuous variables as mean ± SD, non‐parametric continuous variables as median with interquartile ranges and discrete variables as counts or proportions. The association between retraction characteristics and journal IF was analysed with a univariate linear regression model entering reasons for retraction as a dummy variable, followed by Bonferroni post hoc test for multiple comparisons.

## 3 RESULTS

**Figure 1:**
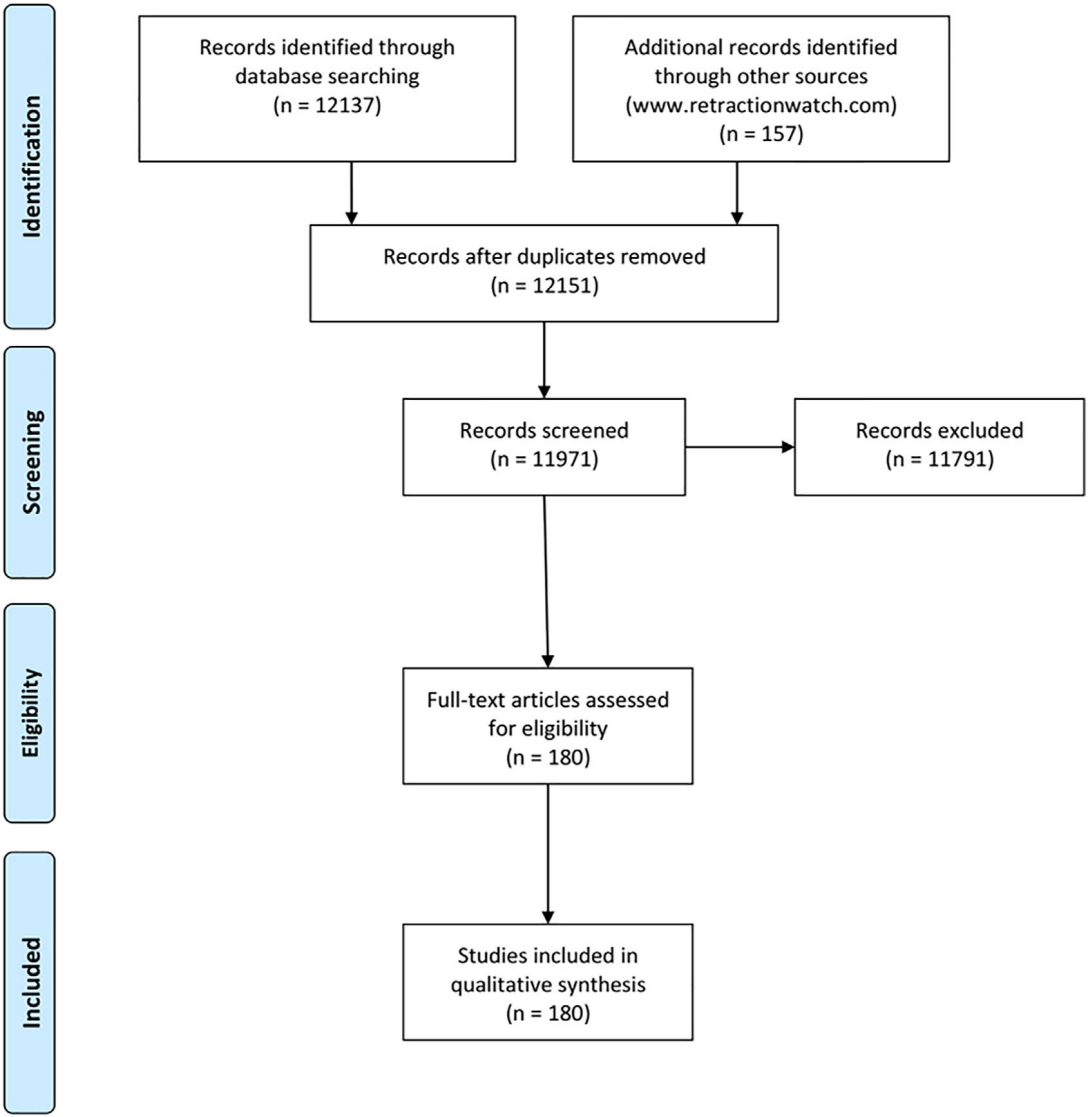
Flowchart of the search process

Electronic search resulted in a total of 12,294 records (12,137 in Pubmed and 157 in Retraction Watch) and, after removing duplicates, a total of 12,151 publications were screened. 11,971 articles were excluded after examination of titles and abstracts and 180 papers were included in the final analysis (inter‐reviewer agreement = 0.98). The results of the electronic search are summarised in Figure 1. The list of retracted articles with their respective retraction notices is reported in the Supporting information (Table S1).

### 3.1 Study characteristics

**Figure 2:**
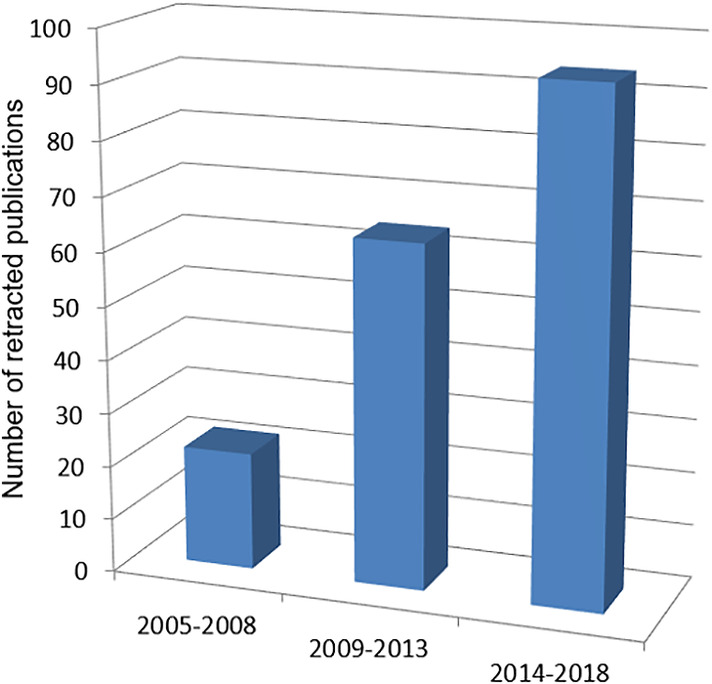
Retracted publications in dentistry over time

The first retracted article was published in 2001 (Sudbø et al., 2001). The last retracted article was published in 2018 (Lin et al., 2018). The first retraction notice was published in 2005 (Nekora‐Azak, 2005), and the last retraction notice was published in 2018 (Alsulaimani, 2018). Mean time elapsed from publication to retraction of the included articles was 2.1 ± 2.4 years (median 1 year). Median year of publication of the retracted papers was 2012. Median year of publication of the retraction notices was 2014. The distribution of the retractions over time is depicted in Figure 2. In vitro studies represented the most frequent typology of retracted article (22.8%), followed by case reports (15.0%) and review articles (14.4%). The complete list of the retracted publications, divided by study design, is reported in Table 1. Oral pathology is the subspecialty with the highest number of retractions (n = 50; 27.8%), followed by implantology (n = 31; 17.2%) and periodontology (n = 23; 12.8%). The complete list is presented in Table 2. In terms of geographical distribution, the majority of retracted research was conducted in Asia (n = 100; 55.6%), with 49 papers written in India (27.2%). Complete data for continent and country are listed in Table 3.

**Table 1:** Study design of retracted publications

Study design	Number	Percentage (%)
In vitro	41	22.8
Case report	27	15.0
Review	26	14.4
Animal study	24	13.3
Prospective	24	13.3
Retrospective	12	6.7
Cross sectional	11	6.1
RCT	8	4.4
Case series	5	2.8
Ex vivo	1	0.6
Letter	1	0.6

Abbreviation: RCT, randomised clinical trial.

**Table 2:** Distribution of retracted publications in dental subspecialties

Discipline	n. publications	%	n. authors
Oral pathology	50	27.8	5.3 ± 3.7
Implantology	31	17.2	4.9 ± 1.7
Periodontology	23	12.8	4.6 ± 2.0
General dentistry	17	9.4	4.3 ± 2.0
Prosthodontics	15	8.3	3.5 ± 1.8
Endodontics	13	7.2	4.1 ± 1.4
Oral surgery	11	6.1	3.9 ± 1.6
Restorative dentistry	11	6.1	4.0 ± 2.2
Gnathology	3	1.7	3.3 ± 1.2
Orthodontics	3	1.7	4.0 ± 1.0
Dental radiology	2	1.1	5.0 ± 1.4
Paediatric dentistry	1	0.6	5.0

*Note:* %: percentage of the total number; number of authors is expressed as mean ± SD.

Abbreviation: n, number.

**Table 3:** Retracted publications in different countries and continents

Nationality	Number	Percentage (%)	Continent	Number	Percentage (%)
India	49	27.2	Asia	100	55.6
Spain	17	9.4	Europa	51	28.3
China	15	8.3	America	19	10.6
Norway	12	6.7	Africa	7	3.9
USA	9	5.0	Various	2	1.1
Iran	8	4,4	Oceania	1	0.6
Brazil	7	3.9			
Germany	7	3.9			
Japan	7	3.9			
Egypt	6	3.3			
Arabia	5	2.8			
Italy	5	2.8			
Turkey	5	2.8			
Others	28	15.6			

620 researchers are listed as authors of the 180 retracted papers included in the present review. Mean number of authors listed in each publication is 4.6 ± 2.5. Thirteen papers were authored by a single researcher, 145 studies had up to six authors and 22 had more than six authors. The distribution of the mean number of authors in the single subspecialties is listed in Table 2. The great majority of the authors (n = 552; 89.0%) are present in only one article. Nevertheless, some authors are present in multiple retracted articles. Two research groups, in particular, authored more than 10 publications retracted for fabricated and/or unreliable data: the first one in Spain (senior researcher José Luis Calvo Guirado – 17 retracted articles), the second in Norway (senior researcher Jon Sudbø – 12 retracted articles).

### 3.2 Retraction characteristics

Author misconduct resulted to be the most common reason for retraction (117 articles; 65.0%), followed by honest scientific errors (22 articles; 12.2%) and publisher‐related issues (19 articles; 10.6%). The reasons for the retraction of 12 articles (6.7%) were not explained in the retraction notice (Abou‐Madina, Ozcan, & Abdelaziz, 2012; Al‐Sukhun, Helenius, Lindqvist, & Thoren, 2007; El Fadl et al., 2011; Jin, Patil, & Sharma, 2014; Lan, Hadj‐Said, Foletti, Massereau, & Chossegros, 2018; Panaite, Klokkevold, & Charles, 2008; Pavel & Pavel, 2010; Saker, El‐Kholany, El‐Gendy, Fadhil, & Maria, 2014; Saker, El‐Kholany, Sakrana, & Maria, 2014; Sumanth, Boaz, & Shetty, 2008; Velleuer et al., 2017; Wang et al., 2008). Detailed description of the reasons for retraction of the included articles is provided in Table 4.

**Table 4:** Retraction characteristics of the included articles

Reason for retraction		Number	Percentage (%)
Misconduct, n = 117 (65.0%)	Plagiarism	40	22.2
	Unreliable data	25	13.9
	Duplication	20	11.1
	Fabricated data	18	10.0
	Overlapping with previous work	12	6.7
	Ethical issues	2	1.1
Scientific error, n = 22 (12.2%)	Methodological flaws	22	12.2
Publisher issues, n = 19 (10.6%)	Double publication	9	5.0
	Unpayment of publication charge	5	2.8
	Publisher error	3	1.7
	Peer review compromised	1	0.6
	Copyright violation	1	0.6
Others, n = 22 (12.2%)	Reason for retraction not reported	12	6.7
	Lack of author permission	8	4.4
	Withdrawn patient consent	1	0.6
	Contributing authors not mentioned	1	0.6

**Figure 3:**
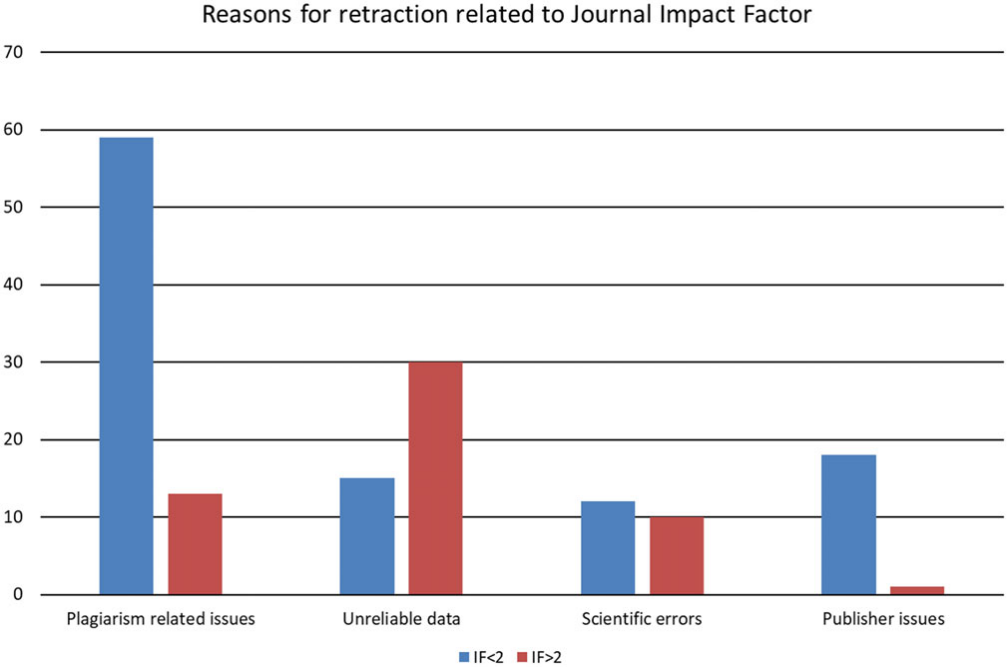
Reason for retraction related with journal impact factor. IF, impact factor

Plagiarism related reasons (duplication, overlapping with previous works) represent the main cause for retraction in journals with IF < 2, while unreliable and/or fabricated data related reasons prevail in journals with IF > 2 (Figure 3). Nevertheless, no statistically significant associations were demonstrated between reasons for retraction and journal IF (*p* = .5496).

140 retracted articles out of 180 (77.8%) are still available in full text on respective journal websites, and 15 of these still available articles (10.7%) present no evident watermark clearly indicating the presence of a retraction (Acharya & Mandal, 2016; Asgary & Eghbal, 2010; Cuoghi, Sella, & de Mendonça, 2010; Dionysopoulos, Koliniotou‐Koumpia, Helvatzoglou‐Antoniades, & Kotsanos, 2013; Ellakwa & El‐Sheikh, 2006; Gulsahi et al., 2010; Khattab, El‐Seify, Shaaban, Radojevic, & Jankovic, 2010; Kurtulmus & Cotert, 2009; Nayyar, Khan, Bafna, Ahmed, & Chaluvaiah, 2014; Ni, Lin, Liu, & Xiao, 2015; Palenik, 2012; Scotti, Cardelli, Baldissara, & Monaco, 2011; Sumanth et al., 2008; Wang et al., 2008). The abstracts of 157 out of 180 retracted publications (87.2%) are still available on Pubmed, but 8 of these (5.1%) present neither a footnote nor any clear indication of the presence of a retraction notice (Agrawal, Singh, Rashmikant, Singh, & Chand, 2011; Dumitrescu, Zetu, & Teslaru, 2008; Ehrlich et al., 2014; Kumar et al., 2010; Maté Sánchez de Val et al., 2015; Rabanal, Bral, & Goldstein, 2007; Wang et al., 2008).

### 3.3 Citations

**Figure 4:**
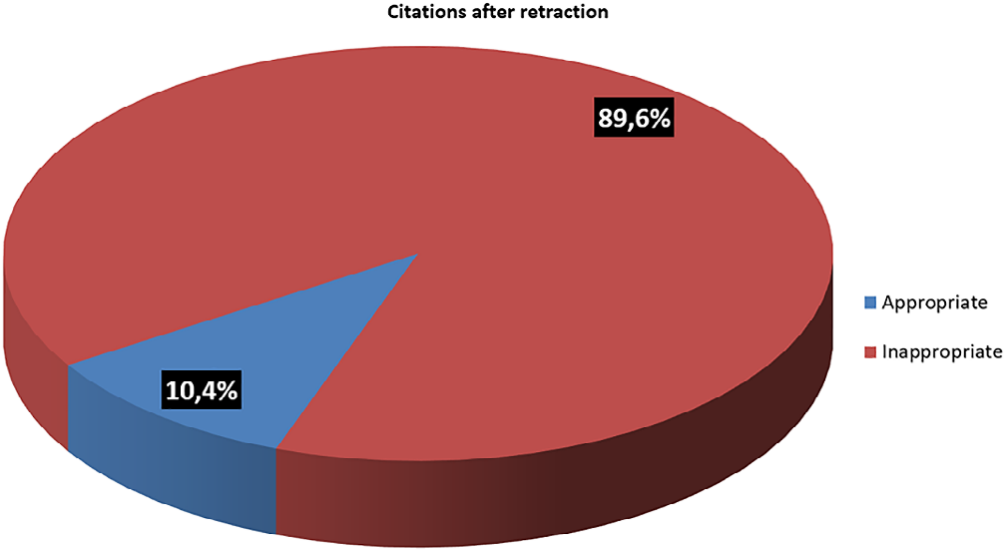
Appropriateness of post‐retraction citations

The articles included in the present systematic review (n = 180) were cited 530 times after their retraction (mean 2.94). Citations in new articles published in the year of the retraction notice and in the subsequent year were excluded from the present analysis in order to eliminate cases of citation by authors potentially unaware of the cited document's retraction. The great majority of the citations of retracted articles (n = 475; 89.6%) did not consider the existence of the retraction notice and treated data from retracted articles as reliable. Only 55 citations of retracted articles (10.4%) were appropriate, reporting information about the retraction in the main text of the article and/or in the bibliography and discussing the questionable value of the findings cited. The distribution of the citations of the retracted articles is graphically depicted in Figure 4.

## 4 DISCUSSION

The retraction of scientific publications has constantly increased in recent years (Steen, 2011; Steen, Casadevall, & Fang, 2013). However, it remains unclear if this trend is mainly the result of increasing intentional scientific misconduct or the result of improvements in the detection of unreliable articles (Cokol et al., 2008), due to the adoption by many journals of the guidelines suggested by the Committee on Publication Ethics (COPE) (2009). Regrettably, it has to be said that many cases of scientific misconduct may remain undetected. A meta‐analysis of surveys conducted among scientists showed that approximately 2% admitted to having fabricated, falsified or modified data or results at least once, and 34% admitted other forms of intentional scientific misconduct, such as graph manipulation and unjustified removal of data from final analyses (Fanelli, 2009). The findings of the present systematic review confirm, in accordance with previous works, that dental literature is currently following the same trend (Faggion Jr et al., 2018; Nogueira, Gonçalves, Leles, Batista, & Costa, 2017). Retractions increased by 47% in the last four‐year period (2014–2018), when compared with 2009–2013 (94 and 64 retracted publications, respectively).

Intentional misconduct resulted by far the most common reason for retraction (65.0%), followed by honest scientific errors (12.2%) and publisher‐related issues (10.6%). These findings are in accordance with the results of recent reviews in both dental (Faggion Jr et al., 2018) and in other medical fields (Chambers, Michener, & Falcone, 2019; Rai & Sabharwal, 2017; Rosenkrantz, 2016). Certain characteristics of retracted articles seem to be related to journal IF, even if these associations do not reach statistical significance. Plagiarism related issues are the most common reason for retraction in journals with IF < 2, while inaccurate or falsified research is the prevailing motivation in journals with IF > 2. This is potentially due to the fact that high quality journals have more efficient tools to detect plagiarism at an early stage of manuscript evaluation and to perform more accurate post‐production control.

The majority of retracted research was conducted in Asia (n = 100; 55.5%), with 49 papers written in India (27.2%). The most common reasons for these retractions are plagiarism related issues. These findings are in accordance with the results reported by Faggion Jr et al. (2018).

The great majority (89%) of the authors of the studies included in the present systematic review appeared in only one retracted paper. Only 10 authors (1.6%), belonging to two different research groups (one in Spain, one in Norway), were present in five or more publications retracted for scientific misconduct and/or presenting unreliable data. These outcomes indicate that retracted publications represent isolated misconduct and negligence more than the result of organised systems persistently ignoring the fundamental principles of ethics and integrity in scientific research.

Unfortunately, retraction does not always end the life of a publication. Citation of retracted papers represents a major issue for the scientific community. This practice leads to the diffusion of false or unreliable information which may be used as premise and foundation for future research, seriously compromising the advancement of science. Retracted articles included in the present review were cited 530 times in the years following the retraction notice (mean 2.96 citations/year). Almost 90% of these citations were inappropriate, treating information from the retracted articles as reliable, as shown in previous studies conducted in dental literature and in different fields of medicine (Budd et al., 1999; Faggion Jr et al., 2018; Grieneisen & Zhang, 2012). Only approximately 10% of the post‐retraction citations made an explicit mention of the retraction and discussed the questionable value of the findings cited. This unfavourable and dangerous situation must be addressed and resolved with an essential active contribution by both researchers and journal editors. Researchers should conduct regular and accurate electronic screening of literature, to ensure that they avoid basing their work on unreliable data. Journal editors should be rigorous and consistent in dealing with retractions. Approximately 78% of the retracted articles included in the present systematic review are still available in full text on their respective journal websites and 11% of these still available retracted articles present no evident watermark clearly indicating the presence of a retraction. Furthermore, even if COPE guidelines recommend that a clear and detailed description of the reasons for retraction should be provided to inform readers (COPE, 2009), it is common to find only generic and unobvious retraction announcements, some of which providing no explanation at all (7% in the present review).

Finally, it is highly desirable that editorial offices perform electronic screening of references citing retracted articles in all newly‐submitted articles, in order to reduce persistence of error in future studies. Cosentino and Veríssimo (2016) proposed that editorial offices utilise a database of retracted articles for cross‐checking purposes to prevent citation of retracted publications.

The system of research evaluation currently adopted in many countries should be seriously reconsidered. Current methods based mainly on the number of works and citations tend to recognise quantity more than quality. Analogously, subject to the same methodologic criteria, the 17th century Dutch master Johannes Vermeer, recognised as having painted only 45 unique masterpieces, would have disappeared entirely from art history, being surpassed and obscured by numerically more prolific contemporary artists producing greater numbers of aesthetically negligible and historically irrelevant paintings.

## CONFLICT OF INTEREST

The authors declare no potential conflict of interest.

## Supporting information


**Data S1.** Supporting information.
