# Supplementary material for: Retracted publications and their citation in dental literature: A systematic review
Source: Clin Exp Dent Res. 2020 Mar 31;6(4):383–90. doi: 10.1002/cre2.292 (PMC7453776; doi:10.1002/cre2.292)
Supplement: Supplementary file 1 — Data S1. Supporting information. [file CRE2-6-383-s001.docx]

Table S1 - LIST OF RETRACTED PUBLICATIONS AND RETRACTION NOTICES

1. Sudbø J, Kildal W, Risberg B, Koppang HS, Danielsen HE, Reith A (2001) DNA content as a prognostic marker in patients with oral leukoplakia. N Engl J Med 344:1270–1278

- Curfman GD, Morrissey S, Drazen JM (2006) Retraction: Sudbø J et al. DNA content as a prognostic marker in patients with oral leukoplakia. N Engl J Med 2001;344:1270-8 and Sudbø J et al. The influence of resection and aneuploidy on mortality in oral leukoplakia. N Engl J Med 2004;350:1405-13. N Engl J Med 355:1927

1. Sudbø J, Bryne M, Johannessen AC, Kildal W, Danielsen HE, Reith A (2001) Comparison of histological grading and large-scale genomic status (DNA ploidy) as prognostic tools in oral dysplasia. J Pathol 194:303–310

- No authors listed (2007) Notice of Retraction: “Comparison of histological grading and large-scale genomic status (DNA ploidy) as prognostic tools in oral dysplasia” (J Pathol 2001;194:303-310). J Pathol 211:109

1. Sudbø J, Ried T, Bryne M, Kildal W, Danielsen H, Reith A (2001) Abnormal DNA content predicts the occurrence of carcinomas in non-dysplastic oral white patches. Oral Oncol 37:558–565

- No authors listed (2007) Retraction notice to “Abnormal DNA content predicts the occurrence of carcinomas in non-dysplastic oral white patches” [Oral Oncol. 37 (2001) 558-565]. Oral Oncol 43:418

1. Uehara A, Sugawara S, Muramoto K, Takada H (2002) Activation of human oral epithelial cells by neutrophil proteinase 3 through protease-activated receptor-2. J Immunol 169:4594–4603

- Sugawara S, Muramoto K (2010) Retraction: Activation of human oral epithelial cells by neutrophil proteinase 3 through protease-activated receptor-2. J Immunol 184:4042

1. Sudbø J, Warloe T, Aamdal S, Reith A, Bryne M (2001) Diagnosis and treatment of oral precancerous lesions. Tidsskr Nor Laegeforen 121:3066-3071

- Warloe T, Aamdal S, Reith A, Bryne M (2006) Retraction of: Diagnostics and treatment of early stages of oral cancer. Tidsskr Nor Laegeforen 126:2287

1. Sudbø J, Reith A (2002) When is an oral leukoplakia premalignant? Oral Oncol. 38:813-814

- No authors listed (2007) Retraction notice to “When is an oral leukoplakia premalignant?” [Oral Oncol. 38 (2002) 813-814]. Oral Oncol 43:419

1. Uehara A, Sugawara S, Watanabe K, Echigo S, Sato M, Yamaguchi T, Takada H (2003) Constitutive expression of a bacterial pattern recognition receptor, CD14, in human salivary glands and secretion as a soluble form in saliva. Clin Diagn Lab Immunol 10:286–292

- No authors listed (2010) Retraction: Constitutive expression of a bacterial pattern recognition receptor, CD14, in human salivary glands and secretion as a soluble form in saliva (Clinical and Diagnostic Laboratory Immunology (2003) 10:2 (286-292)). Clin Vaccine Immunol 17:698

1. Sudbø J, Ristimäki A, Sondresen JE, Kildal W, Boysen M, Koppang HS, Reith A, Risberg B, Nesland JM, Bryne M (2003) Cyclooxygenase-2 (COX-2) expression in high-risk premalignant oral lesions. Oral Oncol 39:497–505

- No authors listed (2007) Retraction notice to “Cyclooxygenase-2 (COX-2) expression in high-risk premalignant oral lesions” [Oral Oncol. 39 (2003) 497-505]. Oral Oncol. 43:420

1. Sudbø J (2003) Chemoprevention of oral cancer. Tidsskr Nor Laegeforen 123:1518-1521

- Haug C (2006) Retraction of: Chemoprevention of oral cancer. Tidsskr Nor Laegeforen 126:2287

1. Gerlach KL, Schwarz A (2003) Load resistance of mandibular angle fracturs treated with a miniplate osteosynthesis. Mund Kiefer Gesichtschir 7:241–245

- No authors listed (2012) Retraction. Belastungsmessungen nach der miniplattenosteosynthese von unterkieferwinkelfrakturen. Oral Maxillofac Surg 16:405

1. Sudbø J, Bryne M, Mao L, Lotan R, Reith A, Kildal W, Davidson B, Søland TM, Lippman SM (2003) Molecular based treatment of oral cancer. Oral Oncol 39:749–758

- No authors listed (2007) Retraction notice to “Molecular based treatment of oral cancer” [Oral Oncol. 39 (2003) 749-758]. Oral Oncol 43:421

1. Nakano M, Fujii Y (2003) Prevention of nausea and vomiting after dental surgery: a comparison of small doses of propofol, droperidol, and metoclopramide. Can J Anaesth 50:1085

- No authors listed (2013) Retraction note to: Prevention of nausea and vomiting after dental surgery: A comparison of small doses of propofol, droperidol, and metoclopramide. Can J Anesth 60:608

1. Sudbø J, Lippman SM, Lee JJ, Mao L, Kildal W, Sudbø A, Sagen S, Bryne M, El-Naggar A, Risberg B, Evensen JF, Reith A (2004) The influence of resection and aneuploidy on mortality in oral leukoplakia. N Engl J Med 350:1405–1413

- Curfman GD, Morrissey S, Drazen JM (2006) Retraction: Sudbø J et al. DNA content as a prognostic marker in patients with oral leukoplakia. N Engl J Med 2001;344:1270-8 and Sudbø J et al. The influence of resection and aneuploidy on mortality in oral leukoplakia. N Engl J Med 2004;350:1405-13. N Engl J Med 355:1927

1. Nekora-Azak A (2004) Temporomandibular disorders in relation to female reproductive hormones: a literature review. J Prosthet Dent 91:491–493

- No authors listed (2005) Retraction notice of: Temporomandibular disorders in relation to female reproductive hormones: A literature review. J Prosthet Dent 94:305

1. Uehara A, Sugawara Y, Sasano T, Takada H, Sugawara S (2004) Proinflammatory cytokines induce proteinase 3 as membrane-bound and secretory forms in human oral epithelial cells and antibodies to proteinase 3 activate the cells through protease-activated receptor-2. J Immunol 173:4179–4189

- Sugawara Y, Sasano T, Sugawara S (2010) Retraction: Proinflammatory cytokines induce proteinase 3 as membrane-bound and secretory forms in human oral epithelial cells and antibodies to proteinase 3 activate the cells through protease-activated receptor-2. J Immunol 184:4044

1. Sudbø J, Samuelsson R, Risberg B, Heistein S, Nyhus C, Samuelsson M, Puntervold R, Sigstad E, Davidson B, Reith A, Berner A (2005) Risk markers of oral cancer in clinically normal mucosa as an aid in smoking cessation counseling. J Clin Oncol 23:1927–1933

- No authors listed (2006) Retraction. J Clin Oncol 24:5621

1. Numazaki M, Fujii Y (2005) Reduction of postoperative emetic episodes and analgesic requirements with dexamethasone in patients scheduled for dental surgery. J Clin Anesth 17:182–186

- No authors listed (2013) Retraction notice to “Reduction of postoperative emetic episodes and analgesic requirements with dexamethasone in patients scheduled for dental surgery” (J Clin Anesth 2005;17:182–86). J Clin Anesth 25:354

1. Sudbø J (2004) Novel management of oral cancer: a paradigm of predictive oncology. Clin Med Res 2:233–242

- Reed KD, Salzman-Scott SA (2007) Retraction - Sudbo J. Novel management of oral cancer: a paradigm of predictive oncology. Clin Med Res 2004;2:233-242. Clin Med Res 5:203

1. Peng JP, Chang HC, Hwang CF, Hung WC (2005) Overexpression of cyclooxygenase-2 in nasopharyngeal carcinoma and association with lymph node metastasis. Oral Oncol 41:903–908

- Peng JP, Chang HC, Hwang CF, Hung WC (2006) Retraction to “Overexpression of cyclooxygenase-2 in nasopharyngeal carcinoma and association with lymph node metastasis” [Oral Oncol. 41 (2005) 903-908]. Oral Oncol 42:653

1. Sudbø J, Lee JJ, Lippman SM, Mork J, Sagen S, Flatner N, Ristimäki A, Sudbø A, Mao L, Zhou X, Kildal W, Evensen JF, Reith A, Dannenberg AJ (2005) Non-steroidal anti-inflammatory drugs and the risk of oral cancer: a nested case-control study. Lancet 366:1359–1366

- Horton R (2006) Retraction - Non-steroidal anti-inflammatory drugs and the risk of oral cancer: a nested case-control study. Lancet 367:382

1. Ellakwa AE, El-Sheikh AM (2006) Effect of chemical disinfectants and repair materials on the transverse strength of repaired heat-polymerized acrylic resin. J Prosthodont 15:300–305

- No authors listed (2013) Retraction statement. Effect of chemical disinfectants and repair materials on the transverse strength of repaired heat-polymerized acrylic resin. J Prosthodont 22:341

1. Huang HH, Lin MC, Lin CC, Lin SC, Hsu CC, Chen FL, Lee SY, Hung CC (2006) Effects of welding pulse energy and fluoride ion on the cracking susceptibility and fatigue behavior of Nd:YAG laser-welded cast titanium joints. Dent Mater J 25:632–640

- No authors listed (2013) Retraction: Effects of welding pulse energy and fluoride ion on the cracking susceptibility and fatigue behavior of Nd:YAG laser-welded cast titanium joints. Dent Mater J 32:195

1. Azadani PN, Jafarimehr E, Shokatbakhsh A, Pourhoseingholi MA, Ghougeghi A (2007) The effect of dental overbite on eustachian tube dysfunction in Iranian children. Int J Pediatr Otorhinolaryngol 71:325–331

- No authors listed (2007) Retraction notice to “The effect of dental overbite on eustachian tube dysfunction in Iranian children” [Int J Pediatr Otorhinolaryngol 2007; 71: 325-31]. Int J Pediatr Otorhinolaryngol 71:1815

1. Ramani P, Chandrasekar T, Anuja N, Muthusekar MR, Sherlin HJ, Kulkarni A (2007) A swelling in the buccal mucosa with intracranial involvement. Oral Surg Oral Med Oral Pathol Oral Radiol Endod 103:308–313

- No authors listed (2008) Retraction notice. Oral Surg Oral Med Oral Pathol Oral Radiol Endod 106:463

1. Al-Sukhun J, Helenius M, Lindqvist C, Thören H (2007) Use of platelet rich plasma (PRP) in the reconstruction of mandibular bony defects: clinical and radiographic follow-up. Br J Oral Maxillofac Surg Jan 6. [Epub ahead of print]

- No authors listed (2008) Withdrawn: Use of platelet rich plasma (PRP) in the reconstruction of mandibular bony defects: clinical and radiographic follow-up. Br J Oral Maxillofac Surg xx:xxx

1. Rabanal A, Bral M, Goldstein G (2007) Management of a patient with severe erosive lichen planus in need of an immediate complete denture: a clinical report. J Prosthet Dent 97:252–255

- No authors listed (xxxx) Retracted: Management of a patient with severe erosive lichen planus in need of an immediate complete denture: A clinical report. J Prosthet Dent xx:xx

1. Quinonez R, Stearns SC (2008) Issues and early evidence for the economic evaluation of the effects of periodontal therapy on pregnancy outcomes. J Periodontol 79:203–206

- Quinonez R, Stearns S (2008) Retracted: Issues and early evidence for the economic evaluation of the effects of periodontal therapy on pregnancy outcomes. J Periodontol 79:771

1. Seyedmajidi M, Faizabadi M (2008) Squamous cell carcinoma of the tongue in a 13-year-old boy. Arch Iran Med 11:341–343

- No authors listed (2010) Retraction. Arch Iran Med 13:174

1. Gopikrishna V, Baweja PS, Venkateshbabu N, Thomas T, Kandaswamy D (2008) Comparison of coconut water, propolis, HBSS, and milk on PDL cell survival. J Endod 34:587–589

- No authors listed (2014) Retracted: Comparison of Coconut Water, Propolis, HBSS, and Milk on PDL Cell Survival 40:290

1. Sumanth KN, Boaz K, Shetty NY (2008) Glass embedded in labial mucosa for 20 years. Indian J Dent Res 19:160–161

- Sumanth KN, Boaz K, Shetty NY (2008) Retraction. Glass embedded in labial mucosa for 20 years. Indian J Dent Res 19:281

1. Wang G, Tai B, Huang C, Bian Z, Shang Z, Wang Q, Song G (2008) Establishing a multidisciplinary PBL curriculum in the School of Stomatology at Wuhan University. J Dent Educ 72:610–615

- No authors listed (2008) Erratum. J Dent Educ 72:e1214

1. Kurtulmus H, Cotert HS (2009) Management of obstructive sleep apnea in an edentulous patient with a combination of mandibular advancement splint and tongue-retaining device: a clinical report. Sleep Breath 13:97–102

- No authors listed (2011) Erratum: Management of obstructive sleep apnea in an edentulous patient with a combination of mandibular advancement splint and tongue-retaining device: A clinical report. Sleep Breath. 15:153

1. Guilleminault C, Quo S, Huynh N T, Li K (2008) Orthodontic expansion treatment and adenotonsillectomy in the treatment of obstructive sleep apnea in prepubertal children. Sleep 31:953-957

- No authors listed (2010) At the request of the corresponding (first) author, the editors of Sleep are retracting the following paper and erratum: Guilleminault C, Quo S, Huynh NT, Li K. Orthodontic expansion treatment and adenotonsillectomy in the treatment of obstructive sleep apnea in prepubertal children. Sleep;31(7):953-957 and Erratum to Guilleminault C, Quo S, Huynh NT, Li K. Orthodontic expansion treatment and adenotonsillectomy in the treatment of obstructive sleep apnea in prepubertal children. Sleep;31(7):953-957, in Sleep 2009;32(1):6. Sleep 33:8

1. de Almeida-Gomes F, Carvalho-Sousa B, Furtado-Leite MC, dos Santos R A, Maniglia-Ferreira C (2007) Effectiveness of single- versus multiple-visit endodontic treatment of two mandibular central incisors from the same patient. Aust Endod J July 21. [Epub ahead of print]

- de Almeida-Gomes F, Carvalho-Sousa B, Furtado-Leite MC, dos Santos RA, Maniglia-Ferreira C (2008) Retraction: Effectiveness of single- versus multiple-visit endodontic treatment of two mandibular central incisors from the same patient. Aust Endod J 34:76

1. Naqvi N, Naqvi R, Wong C, Pearce S (2008) A novel observation of pubic osteomyelitis due to Streptococcus viridans after dental extraction: a case report. J Med Case Rep 2:255

- Naqvi N, Naqvi R, Wong C, Pearce S (2009) Retraction: A novel observation of pubic osteomyelitis due to Streptococcus viridans after dental extraction: a case report. J Med Case Rep 3:122

1. Aggarwal V, Logani A, Shah N (2008) The evaluation of computed tomography scans and ultrasounds in the differential diagnosis of periapical lesions. J Endod 34:1312–1315

- No authors listed (2017) Retracted. J. Endod. 43:833

1. Panaite D, Klokkevold P, Charles A (2008) The peri-implant papilla: realities on papilla preservation and reformation. J Calif Dent Assoc 36:851–867

- Felsenfeld AL, Carney KK (2009) Retraction: Panaite D, Klokkevold P, and Charles A. The peri-implant papilla: realities on papilla preservation and reformation. J Calif Dent Assoc 36(11):851-67, 2008. J Calif Dent Assoc 37:71

1. Nikitakis NG, Scheper MA, Papanikolaou VS, Sauk JJ (2009) The oncogenic effects of constitutive Stat3 signaling in salivary gland cancer cells are mediated by survivin and modulated by the NSAID sulindac. Oral Surg Oral Med Oral Pathol Oral Radiol Endod 107:826–836

- Nikitakis NG, Scheper MA, Papanikolaou VS, Sauk JJ (2014) Retracted: The oncogenic effects of constitutive Stat3 signaling in salivary gland cancer cells are mediated by survivin and modulated by the NSAID sulindac (Oral Surgery, Oral Medicine, Oral Pathology and Oral Radiology). Oral Surg. Oral Med. Oral Pathol. Oral Radiol. 118:746

1. Dumitrescu AL, Zetu L, Teslaru S (2008) Metabolic syndrome and periodontal diseases. Rom J Intern Med 46:207–212

- Editorial Board of Romanian Journal of Internal Medicine. (2016) Retraction of six papers from the Romanian Journal of Internal Medicine. Rom J Intern Med 54:256

1. Yazdi I, Seyedmajidi M, Foroughi R (2009) Desmoplastic ameloblastoma (a hybrid variant): report of a case and review of the literature. Arch Iran Med 12:304–308

- No authors listed (2010) Retraction. Arch Iran Med 13:174

1. Seyedmajidi M, Feizabadi M (2009) Peripheral calcifying odontogenic cyst. Arch Iran Med 12:309–312

- No authors listed (2010) Retraction. Arch Iran Med 13:174

1. Nosrati K, Seyedmajidi M (2009) Ameloblastomatous calcifying odontogenic cyst: a case report of a rare histologic variant. Arch Iran Med 12:417–420

- No authors listed (2010) Retraction. Arch Iran Med 13:174

1. Khattab AM, El-Seify ZA, Shaaban A, Radojevic D, Jankovic I (2010) Sevoflurane-emergence agitation: effect of supplementary low-dose oral ketamine premedication in preschool children undergoing dental surgery. Eur J Anaesthesiol 27:353–358

- No authors listed (2011) Sevoflurane-emergence agitation: effect of supplementary low-dose oral ketamine premedication in preschool children undergoing dental surgery: Retraction. Eur J Anaesthesiol 28,310

1. Topcu FT, Erdemir U, Sahinkesen G, Mumcu E, Yildiz E, Uslan I (2010) Push-out bond strengths of two fiber post types bonded with different dentin bonding agents. J Biomed Mater Res B Appl Biomater 93:359–366

- No authors listed (2012) Retraction: Push-out bond strengths of two fiber post types bonded with different dentin bonding agents. J Biomed Mater Res B Appl Biomater 100:1458

1. Kumar S, Atray D, Paiwal D, Balasubramanyam G, Duraiswamy P, Kulkarni S (2010) Dental unit waterlines: source of contamination and cross-infection. J Hosp Infect 74:99–111

- No authors listed (2011) Retraction notice to “Dental unit waterlines: source of contamination and cross-infection” [J Hosp Infect 74 (2010) 99-111]. J Hosp Infect 78:340

1. Cuoghi OA, Sella RC, de Mendonça MR (2010) Mesiodistal angulations of the mandibular canines, premolars and molars with or without the presence of third molars. Eur J Orthod 32:472–476

- No authors listed (2016) Retraction. Eur. J. Orthod. 38:225

1. Panagiotopoulou O, Curtis N, O’ Higgins P, Cobb SN (2010) Modelling subcortical bone in finite element analyses: A validation and sensitivity study in the macaque mandible. J Biomech 43:1603–1611

- No authors listed (2015) Retraction notice to "Modelling subcortical bone in finite element analyses: a validation and sensitivity study in the macaque mandible" [J. Biomech. 43 (2010) 1603-1611]. J Biomech 48:1233

1. Pavel L, Pavel S (2010) Usefulness of micronutrients in the treatment of periodontitis. Ned Tijdschr Tandheelkd 117:103–106

- No authors listed (2013) Retraction: Usefulness of micronutrients in the treatment of periodontitis. Ned Tijdschr Tandheelkd 120:4

1. El Fadl KA, Ragy N, El Batran M, Kassem N, Nasry SA, Khalifa R, Sedrak H, Isenovic ER (2011) Periodontitis and cardiovascular disease: Floss and reduce a potential risk factor for CVD. Angiology 62:62–67

- No authors listed (2011) Retraction: Periodontitis and cardiovascular disease: Floss and reduce a potential risk factor for CVD. Angiology 62:352

1. Asgary S, Eghbal MJ (2010) A clinical trial of pulpotomy vs. root canal therapy of mature molars. J Dent Res 89:1080–1085

- Giannobile WV (2011) Retraction. A clinical trial of pulpotomy vs. root canal therapy of mature molars. J Dent Res 89:1080-1085. J Dent Res 90:1145

1. Panagiotopoulou O, Kupczik K, Cobb SN (2011) The mechanical function of the periodontal ligament in the macaque mandible: a validation and sensitivity study using finite element analysis. J Anat 218:75–86

- No authors listed (2015) Retraction. ‘The mechanical function of the periodontal ligament in the macaque mandible: a validation and sensitivity study using ﬁnite element analysis’ by O. Panagiotopoulou, K. Kupczik and S.N. Cobb. J Anat 226:498

1. Gulsahi A, Paksoy CS, Ozden S, Kucuk NO, Cebeci AR, Genc Y (2010) Assessment of bone mineral density in the jaws and its relationship to radiomorphometric indices. Dentomaxillofac Radiol 39:284–289

- No authors listed (2013) Notice of redundant publication. Dentomaxillofac Radiol 42:20139012

1. Kim JK, Cho JH, Lee YJ, Kim CH, Bae JH, Lee JG, Yoon JH (2010) Anatomical variability of the maxillary artery: findings from 100 Asian cadaveric dissections. Arch Otolaryngol Head Neck Surg 136:813–818

- Kim JK, Cho JH, Lee YJ, Kim CH, Bae JH, Lee JG, Yoon JH (2012) Notice of retraction: duplicate data reported in “Anatomical variability of the maxillary artery: findings from 100 Asian cadaveric dissections” (Arch Otolaryngol Head Neck Surg. 2010;136[8]:813-818.) Arch Otolaryngol Head Neck Surg 138:525

1. Alves da Cunha T de M, Correia de Araújo RP, Barbosa da Rocha PV, Pazos Amoedo RM (2012) Comparison of fit accuracy between Procera custom abutments and three implant systems. Clin Implant Dent Relat Res 14:772–777

- No authors listed (2016) Notice of Withdrawal: “Comparison of fit accuracy between Procera custom abutments and three implant systems” by Tiago de Morais Alves da Cunha, Roberto Paulo Correia de Araújo, Paulo Vicente Barbosa da Rocha and Rosa Maria Pazos Amoedo. Clin Implant Dent Relat Res 18:421

1. Deng T, Wang L, Lv J, Pang J, Liu B, Du Y, Ke J (2011) Association of three bacterial species and periodontal status in Chinese adults: an epidemiological approach. J Clin Microbiol 49:184–188

- No authors listed (2011) Retraction: Association of three bacterial species and periodontal status in Chinese adults: an epidemiological approach. J Clin Microbiol 49:2082

1. Ramírez-Fernández MP, Calvo-Guirado JL, Arcesio Delgado-Ruiz R, Maté-Sánchez Del Val JE, Gómez-Moreno G, Guardia J (2011) Experimental model of bone response to xenografts of bovine origin (Endobon): a radiological and histomorphometric study. Clin Oral Implants Res 22:727–734

- No authors listed (2018) Retraction. Clin. Oral Implants Res 29:665

1. Ramírez-Fernández M, Calvo-Guirado JL, Delgado-Ruiz RA, Maté-Sánchez Del Val JE, Vicente-Ortega V, Meseguer-Olmos L (2011) Bone response to hydroxyapatites with open porosity of animal origin (porcine [OsteoBiol mp3] and bovine [Endobon]): a radiological and histomorphometric study. Clin Oral Implants Res 22:767–773

- No authors listed (2018) Retraction. Clin Oral Implants Res 29:666

1. Barone A, Ricci M, Calvo-Guirado JL, Covani U (2011) Bone remodelling after regenerative procedures around implants placed in fresh extraction sockets: an experimental study in Beagle dogs. Clin Oral Implants Res 22:1131–1137

- No authors listed (2018) Retraction. Clin Oral Implants Res 29:539

1. Agrawal KK, Singh SV, Rashmikant US, Singh RD, Chand P (2011) Prosthodontic rehabilitation in Sjogren’s syndrome with a simplified palatal reservoir: two year follow up. J Prosthodont Res 55:248-251

- No authors listed (2018) Withdrawn: Prosthodontic rehabilitation in Sjogren's syndrome with a simplified palatal reservoir: two year follow up. J Prosthodont Res 55:248

1. Negri B, Calvo-Guirado JL, Pardo-Zamora G, Ramírez-Fernández MP, Delgado-Ruíz RA, Muñoz-Guzón F (2012) Peri-implant bone reactions to immediate implants placed at different levels in relation to crestal bone. Part I: a pilot study in dogs. Clin Oral Implants Res 23:228–235

- No authors listed (2018) Retraction. Clin Oral Implants Res 29:668

1. Bhola R, Su F, Krull CE (2011) Functionalization of titanium based metallic biomaterials for implant applications. J Mater Sci Mater Med 22:1147–1159

- Best S, Planell JA (2011) Retraction note to: Functionalization of titanium based metallic biomaterials for implant applications. J Mater Sci Mater Med 22:2833

1. Calvo-Guirado JL, Delgado-Ruíz RA, Ramírez-Fernández MP, Maté-Sánchez JE, Ortiz-Ruiz A, Marcus A (2012) Histomorphometric and mineral degradation study of Ossceram: a novel biphasic B-tricalcium phosphate, in critical size defects in rabbits. Clin Oral Implants Res 23:667–675

- No authors listed (2018) Retraction. Clin Oral Implants Res 29:669

1. Shetty DC, Urs AB, Ahuja P, Sahu A, Manchanda A, Sirohi Y (2011) Mineralized components and their interpretation in the histogenesis of peripheral ossifying fibroma. Indian J Dent Res 22:56–61

- No authors listed (2012) Retraction notices. Indian J Dent Res 23:682

1. Tanaka T, Tanaka M, Tanaka T (2011) Oral carcinogenesis and oral cancer chemoprevention: a review. Patholog Res Int 2011:431246

- Pathology Research International (2016) Retracted: Oral Carcinogenesis and Oral Cancer Chemoprevention: A Review. Patholog Res Int 2016:9267585

1. Yang X, Walboomers XF, Bian Z, Jansen JA, Fan M (2011) Effects of pro-inflammatory cytokines on mineralization potential of rat dental pulp stem cells. J Tissue Eng Regen Med Jul 11. [Epub ahead of print]

- No authors listed (2011) Retracted: Effects of pro-inflammatory cytokines on mineralization potential of rat dental pulp stem cells. J Tissue Eng Regen Med 5:759

1. Cai C, Rodepeter FR, Rossmann A, Teymoortash A, Lee JS, Quint K, Di Fazio P, Ocker M, Werner JA, Mandic R (2011) Nef from SIV(mac239) decreases proliferation and migration of adenoid-cystic carcinoma cells and inhibits angiogenesis. Oral Oncol 47:847–854

- No authors listed (2012) Retraction notice to "Nef from SIV(mac239) decreases proliferation and migration of adenoid-cystic carcinoma cells and inhibits angiogenesis" [OO 47 (2011) 847–854]. Oral Oncol 48:95

1. Shetty DC, Urs AB, Manchanda A, Sirohi Y (2011) A color contrast aided density imaging technique to differentiate between dental hard tissues and its relevance. Indian J Dent Res 22:266–269

- No authors listed (2012) Retraction notices. Indian J Dent Res 23:682

1. Koduganti RR, Sandeep N, Guduguntla S, Chandana Gorthi VS (2011) Probiotics and prebiotics in periodontal therapy. Indian J Dent Res 22:324–330

- No authors listed (2012) Retraction notices. Indian J Dent Res 23:682

1. El Chaar E, Bettach R (2011) Immediate placement and provisionalization of implant-supported, single-tooth restorations: a retrospective study. J Oral Implantol Sep 9. [Epub ahead of print]

- No authors listed (2012) Retraction. Immediate placement and provisionalization of implant-supported, single-tooth restorations: a retrospective study. J Oral Implantol 38:434

1. Goenka P, Dutta S, Marwah N (2011) Biological approach for management of anterior tooth trauma: triple case report. J Indian Soc Pedod Prev Dent 29:180–186

- No authors listed (2012) Retraction notice. J Indian Soc Pedod Prev Dent 30:282

1. Yang X, Zhang S, Pang X, Fan M (2012) Pro-inflammatory cytokines induce odontogenic differentiation of dental pulp-derived stem cells. J Cell Biochem 113:669–677

- No authors listed (2012) Retraction: Pro-inflammatory cytokines induce odontogenic differentiation of dental pulp-derived stem cells. X Yang, S Zhang, X Pang, and M Fan. J Cell Biochem 113:2796

1. Scotti R, Cardelli P, Baldissara P, Monaco C (2011) Clinical fitting of CAD/CAM zirconia single crowns generated from digital intraoral impressions based on active wavefront sampling. J Dent Oct 17. [Epub ahead of print]

- Scotti R, Cardelli P, Baldissara P, Monaco C (2011) Withdrawn: Clinical fitting of CAD/CAM zirconia single crowns generated from digital intraoral impressions based on active wavefront sampling. J Dent Oct 17. [Epub ahead of print]

1. Negri B, Calvo-Guirado JL, Ramírez-Fernández MP, Maté Sánchez-de Val J, Guardia J, Muñoz-Guzón F (2012) Peri-implant bone reactions to immediate implants placed at different levels in relation to crestal bone. Part II: a pilot study in dogs. Clin Oral Implants Res 23:236–244

- No authors listed (2018) Retraction. Clin. Oral Implants Res 29:667

1. Shetty DC, Urs AB, Rai HC, et al (2010) Case series on vascular malformation and their review with regard to terminology and categorization. Contemp Clin Dent 1:259–262

- No authors listed (2012) Retraction notice. Contemp Clin Dent 3:392

1. Zhang Q, Witter DJ, Bronkhorst EM, Creugers NH (2011) Chewing ability in an adult Chinese population. Clin Oral Investig Dec 2. [Epub ahead of print]

- No authors listed (2012) Retraction. Chewing ability in an adult Chinese population. Clin Oral Investig 16:1511

1. Rajendran R, Deepthi K, Nooh N, Anil S (2011) α4β1 integrin-dependent cell sorting dictates T-cell recruitment in oral submucous fibrosis. J Oral Maxillofac Pathol 15:272–277

- No authors listed (2012) Retraction notice. J Oral Maxillofac Pathol 16:342

1. Deshmukh SP, Radke UM (2012) Translation and validation of the Hindi version of the Geriatric Oral Health Assessment Index. Gerodontology 29:e1052-1058

- MacEntee M (2012) Retraction. Translation and validation of the Hindi version of the Geriatric Oral Health Assessment Index. Gerodontology 29:243

1. Yang X, Zhang S, Pang X, Fan M (2012) Mineralized tissue formation by bone morphogenetic protein-7-transfected pulp stem cells. J Endod 38:170–176

- No authors listed (2012) Retraction notice to mineralized tissue formation by bone morphogenetic protein-7-transfected pulp stem cells: J Endod 38(2012)170-176. J Endod 38:868

1. Abou-Madina MM, Özcan M, Abdelaziz KM (2012) Influence of resin cements and aging on the fracture resistance of IPS e.max press posterior crowns. Int J Prosthodont 25:33–35

- No authors listed (2012) Article withdrawn. "Influence of resin cements and aging on the fracture resistance of IPS e.max press posterior crowns". Int J Prosthodont 25:109

1. Moll D, Yildirim M, Spiekermann H, Wolfart S (2012) Telescopic crown-retained removable partial dentures on teeth and implants: an 8- to 9-year prospective randomized clinical trial. Clin Oral Implants Res Feb 13. [Epub ahead of print]

- No authors listed (2012) Retracted: Telescopic crown-retained removable partial dentures on teeth and implants: an 8- to 9-year prospective randomized clinical trial. Clin Oral Implants Res 23:895

1. Pohlenz P, Atac A, Catala.Lehnen P, Khakpour P, Li L, Klatt J, Schmelzle R (2012) Donor site morbidity of the vascularized fibula: the Hamburg experience. Clin Oral Investig Mar 16. [Epub ahead of print]

- No authors listed (2012) Retracted article: Donor site morbidity of the vascularized fibula: the Hamburg experience. Clin Oral Investig 16:1333

1. Pieri F, Nicoli Aldini N, Fini M, Marchetti C, Corinaldesi G (2012) Rehabilitation of the atrophic posterior maxilla using short implants or sinus augmentation with simultaneous standard-length implant placement: a 3-year randomized clinical trial. Clin Implant Dent Relat Res Mar 15. [Epub ahead of print]

- No authors listed (2012) Retraction. Rehabilitation of the atrophic posterior maxilla using short implants or sinus augmentation with simultaneous standard-length implant placement: a 3-year randomized clinical trial. Clin Implant Dent Relat Res 14:924

1. Gill JS, Gill S, Bhardwaj A, Grover HS (2012) Oral haemangioma. Case Rep Med 2012:347939

- Case Reports in Medicine (2014) Retracted: oral haemangioma. Case Rep Med 2014:942352

1. Mahmoudi M, Saidi A, Gandjalikhan Nassab SA, Hashemipour MA (2012) A three-dimensional finite element analysis of the effects of restorative materials and post geometry on stress distribution in mandibular molar tooth restored with post-core crown. Dent Mater J 31:171–179

- No authors listed (2014) Retraction: A three-dimensional finite element analysis of the effects of restorative materials and post geometry on stress distribution in mandibular molar tooth restored with post-core crown. Dent Mater J 33:147

1. El-Sheikh AM, Shihabuddin OF, Ghoraba SMF (2012) A prospective study of early loaded single implant-retained mandibular overdentures: preliminary one-year results. Int J Dent 2012:236409

- El-Sheikh AM, Shihabuddin OF, Ghoraba SMF (2013) Retracted: A prospective study of early loaded single implant-retained mandibular overdentures: Preliminary one-year results. Int J Dent 2013:310726

1. Bansal S, Shetty S, Bablani D, Kulkarni S, Kumar V, Desai R (2011) Florid osseous dysplasia. J Oral Maxillofac Pathol 15:197–200

- No authors listed (2012) Retraction notice. J Oral Maxillofac Pathol 16:353

1. Palenik CJ (2012) The effect of long-term disinfection on clinical contact surfaces. J Am Dent Assoc 143:472–477

- No authors listed (2012) Notice of retraction. J Am Dent Assoc 143:968–969

1. Subramaniam P, Prashanth P (2012) Prevalence of early childhood caries in 8 - 48 month old preschool children of Bangalore city, South India. Contemp Clin Dent 3:15-21

- No authors listed (2014) Retraction notice. Contemp Clin Dent 5:169

1. Banerjee S, Chakraborty N, Singh R, Gupta T (2012) Full-mouth rehabilitation of a patient with severe attrition using the Hobo twin-stage procedure. Contemp Clin Dent 3:103-107

- No authors listed (2013) Retraction notice. Contemp Clin Dent 4:396

1. Batista Rd, Rosetti EP, Zandonade E, Roelke LH, Vettore MV, Oliveira AE (2012) Association between periodontal disease and subclinical atherosclerosis: the ELSA-Brasil study. Cad Saude Publica 28:965–976

- Batista Rd, Rosetti EP, Zandonade E, Roelke LH, Vettore MV, Oliveira A (2012) Retraction: Association between periodontal disease and subclinical atherosclerosis: the ELSA-Brasil study [Cad Saúde Pública 2012; 28(5): 965-976]. Cad Saude Publica 28:1613

1. Gathecha G, Makokha A, Wanzala P, Omolo J, Smith P (2012) Dental caries and oral health practices among 12 year old children in Nairobi West and Mathira West Districts, Kenya. Pan Afr Med J 12:42

- No authors listed (2015) Retraction: Redundant Publication of the article Dental caries and oral health practices among 12 year old children in Nairobi West and Mathira West Districts, Kenya. Gladwell Gathecha et al. The Pan African Medical Journal. 2012;12:42. Pan Afr Med J 22:233

1. Barros SE, Janson G, Chiqueto K, Ferreira ES, Janson M (2012) Selective use of hand and forearm muscles during bone screw insertion: A natural torque meter. J Oral Maxillofac Surg 70:e598-607

- No authors listed (2013) Retraction: Selective use of hand and forearm muscles during bone screw insertion: a natural torque meter. J Oral Maxillofac Surg 71:981

1. Santander S, Alcaine C, Lyahyai J, Pérez MA, Rodellar C, Doblaré M, Ochoa I (2012) In vitro osteoinduction of human mesenchymal stem cells in biomimetic surface modified titanium alloy implants. Dent Mater J 31:843–850

- No authors listed (2014) Retraction: In vitro osteoinduction of human mesenchymal stem cells in biomimetic surface modified titanium alloy implants. Dent Mater J 33:148

1. Boonanantanasarn K, Janebodin K, Suppakpatana P, Arayapisit T, Rodsutthi JA, Chunhabundit P, Boonanuntanasarn S, Sripairojthikoon W (2012) Morinda citrifolia leaves enhance osteogenic differentiation and mineralization of human periodontal ligament cells. Dent Mater J 31:863–871

- No authors listed (2014) Retraction: Morinda citrifolia leaves enhance osteogenic differentiation and mineralization of human periodontal ligament cells. Dent Mater J 33:149

1. Kumar NSM, Prabu PS, Prabu N, Rathinasamy S (2012) Sealing ability of lateral condensation, thermoplasticized gutta-percha and flowable gutta-percha obturation techniques: A comparative in vitro study. J Pharm Bioallied Sci 4:S131-135

- No authors listed (2015) Sealing ability of lateral condensation, thermoplasticized gutta-percha and flowable gutta-percha obturation techniques: A comparative in vitro study: Retraction. J Pharm Bioallied Sci 7:S830

1. Rastogi P (2012) Emergence of cancer stem cells in head and neck squamous cell carcinoma: A therapeutic insight with literature review. Dent Res J (Isfahan) 9:239–244

- No authors listed (2015) Emergence of cancer stem cells in head and neck squamous cell carcinoma: A therapeutic insight with literature review: Retraction. Dent Res J (Isfahan). 12:498

1. Subramani T, Senthilkumar K, Periasamy S, Rao S (2013) Expression of angiotensin II and its receptors in cyclosporine-induced gingival overgrowth. J Periodontal Res 48:386–391

- No authors listed (2014) Retraction: “Expression of angiotensin II and its receptors in cyclosporine-induced gingival overgrowth” by T. Subramani, K. Senthilkumar, S. Periasamy, S. Rao. J Periodontal Res 49:275

1. Bottini LP, Ricci L, Piattelli A, Perrotti V, Iezzi G (2012) Bucco-lingual crestal bone changes around implants immediately placed in fresh extraction sockets in association or not with porcine bone: a non-blinded randomized controlled trial in humans. J Periodontol Oct 29. [Epub ahead of print]

- Bottini LP, Ricci L, Piattelli A, Perrotti V, Iezzi G (2017) Retracted: Bucco‐lingual crestal bone changes around implants immediately placed in fresh extraction sockets in association or not with porcine bone: a non‐blinded randomized controlled trial in humans (J Periodontol October 29, 2012 [published online ahead of print]; doi: 10.1902/jop.2012.120396). J Periodontol 88:1374

1. Pedaballi P, Sundaram R, Ramachandran M (2012) Prevalence of gingival enlargement secondary to calcium channel blockers in patients with cardiovascular diseases. J Indian Soc Periodontol 16:430–435

- No authors listed (2012) Notice of retraction. J Indian Soc Periodontol 16:vi

1. Baghele ON (2012) Buccinator muscle repositioning. J Indian Soc Periodontol 16:456–460

- No authors listed (2012) Notice of retraction. J Indian Soc Periodontol 16:vi

1. Jacinto-Alemán LF, García-Carrancá A, Leyba-Huerta ER, Zenteno-Galindo E, Jiménez-Farfán MD, Hernández-Guerrero JC (2013) erbB expression changes in ethanol and 7,12- dimethylbenz (a)anthracene-induced oral carcinogenesis.. Med Oral Patol Oral Cir Bucal 18:e325-331

- No authors listed (2014) Retraction: erbB expression changes in ethanol and 7,12- dimethylbenz (a)anthracene-induced oral carcinogenesis. Med Oral Patol Oral Cir Bucal. 2013 Mar 1;18(2):e325-31. Med Oral Patol Oral Cir Bucal 19:e98

1. Kulkarni VK, Deshmukh J, Banda NR, Banda VR (2012) Odontomas--silent tormentors of teeth eruption, shedding and occlusion. BMJ Case Rep 2012:Dec 14

- No authors listed (2013) Retraction. Odontomas--silent tormentors of teeth eruption, shedding and occlusion. BMJ Case Rep 2013:Aug 29

1. Kumar S, Arora A, Yadav R (2012) Prosthetic rehabilitation of edentulous patient with limited oral access: A clinical report. Contemp Clin Dent 3:349-351

- No authors listed (2016) Retraction: Prosthetic rehabilitation of edentulous patient with limited oral access: A clinical report. Contemp Clin Dent 7:124

1. Calvo-Guirado JL, Boquete-Castro A, Negri B, Delgado Ruiz R, Gómez-Moreno G, Iezzi G. (2014) Crestal bone reactions to immediate implants placed at different levels in relation to crestal bone. A pilot study in Foxhound dogs. Clin Oral Implants Res 25:344–351

- No authors listed (2018) Retraction. Clin Oral Implants Res 29:814

1. Sujesh M, Rangarajan V, Ravi Kumar C, Sunil Kumar G (2012) Stem cell mediated tooth regeneration: new vistas in dentistry. J Indian Prosthodont Soc 12:1–7

- Sujesh M, Rangarajan V, Ravi Kumar C, Sunil Kumar G (2014) Retraction note to: Stem cell mediated tooth regeneration: new vistas in dentistry. J Indian Prosthodont Soc 14:351

1. Vyawahare S, Banda NR, Barodiya A, Banda VR (2013) A rare occurrence of peripheral ossifying fibroma in the first decade of life and its management. BMJ Case Rep 2013:Mar 15

- No authors listed (2013) Retraction. A rare occurrence of peripheral ossifying fibroma in the first decade of life and its management. BMJ Case Rep. 2013:Aug 23

1. Dionysopoulos D, Koliniotou-Koumpia E, Helvatzoglou-Antoniades M, Kotsanos N (2013) Fluoride release and recharge abilities of contemporary fluoride-containing restorative materials and dental adhesives. Dent Mater J 32:296–304

- No authors listed (2015) Retraction: Fluoride release and recharge abilities of contemporary fluoride-containing restorative materials and dental adhesives. Dent Mater J 34:410

1. Barodiya A, Banda NR, Banda VR, Vyawahare S (2013) Maxillary adenomatoid odontogenic tumour. BMJ Case Rep 2013:Jun14

- No authors listed (2013) Retraction. Maxillary adenomatoid odontogenic tumour. BMJ Case Rep 2013:Aug 23

1. Moghe S, Saini N, Moghe A (2012) Platelet-rich plasma in periodontal defect treatment after extraction of impacted mandibular third molars. Natl J Maxillofac Surg 3:139–143

- No authors listed (2014) Platelet-rich plasma in periodontal defect treatment after extraction of impacted mandibular third molars: Retraction. Natl J Maxillofac Surg 5:254

1. Vashisht R, Indira R, Ramachandran S, Kumar A, Srinivasan MR (2013) Role of casein phosphopeptide amorphous calcium phosphate in remineralization of white spot lesions and inhibition of Streptococcus mutans? J Conserv Dent 16:342–346

- No authors listed (2016) Retraction: Role of casein phosphopeptide amorphous calcium phosphate in remineralization of white spot lesions and inhibition of Streptococcus mutans? J. Conserv. Dent. 19:198

1. Chopra R, Mathur S (2013) Probiotics in dentistry: A boon or sham. Dent Res J (Isfahan) 10:302–306

- No authors listed (2014) Retraction notice. Dent Res J (Isfahan) 11:308

1. Calvo-Guirado JL, Ramírez-Fernández MP, Delgado-Ruíz RA, Maté-Sánchez JE, Velasquez P, de Aza PN (2014) Influence of biphasic β-TCP with and without the use of collagen membranes on bone healing of surgically critical size defects. A radiological, histological, and histomorphometric study. Clin Oral Implants Res 25:1228–1238

- No authors listed (2018) Retraction. Clin Oral Implants Res 29:663

1. Thapar R, Choudhry S, Sinha A, Bali R, Shukla D (2013) Pink tooth phenomenon: an enigma? J Forensic Leg Med 20:912–914

- No authors listed (2016) Retraction notice to: Pink tooth phenomenon: An enigma? [J Forensic Leg Med 20(7) (October 2013) 912-914]. J. Forensic Leg Med 38:122

1. Ali SM, Nair R, Shetty RS, Karthikeyan M, Nair A, Sial S (2013) Influence of post fit and post length on fracture resistance: an in vitro study. J Contemp Dent Pract 14:496–500

- No authors listed (2013) Retraction notice to: influence of post fit and post length on fracture resistance: an in vitro study. J Contemp Dent Pract 2013;14(3):496-500. J Contemp Dent Pract 14:1008

1. Aziz Aly LA, Menoufy HE, Ragae A, Rashed LA, Sabry D (2012) Adipose stem cells as alternatives for bone marrow mesenchymal stem cells in oral ulcer healing. Int J Stem Cells 5:104–114

- Aziz Aly LA, Menoufy HE, Ragae A, Rashed LA, Sabry D (2014) Adipose stem cells as alternatives for bone marrow mesenchymal stem cells in oral ulcer healing. Int J Stem Cells 7:167

1. Hiyama T, Ozeki N, Mogi M, Yamaguchi H, Kawai R, Nakata K, Kondo A, Nakamura H (2013) Matrix metalloproteinase-3 in odontoblastic cells derived from Ips cells: unique proliferation response as odontoblastic cells derived from ES cells. PLoS One 8:e83563

- PLoS One Editors (2018) Retraction: Matrix metalloproteinase-3 in odontoblastic cells derived from Ips cells: unique proliferation response as odontoblastic cells derived from ES cells. PLoS One 13: e0198542

1. Pendyala G, Joshi S, Chaudhari S, Gandhage D (2013) Links demystified: Periodontitis and cancer. Dent Res J (Isfahan) 10:704–712

- No authors listed (2014) Retraction notice. Dent Res J (Isfahan) 11:308

1. Venkateswar Rao G, Kanthem RK, Cherukuri G, Korlepara R, Kumari G (2012) Oro-facial cysticercosis: a rare presentation. J Parasit Dis Oct 17. [Epub ahead of print]

- Venkateswar Rao G, Kanthem RK, Cherukuri G, Korlepara R, Kumari G (2013) Retracted article: Oro-facial cysticercosis: a rare presentation. J Parasit Dis 37:294

1. Mansuri S, Abdulkhayum AM, Gazal G, Hussain MA (2013) Treatment of mandibular angle fracture with a 2mm, 3 dimensional rectangular grid compression miniplates: A prospective clinical study. J Int Oral Health 5:93–100

- No authors listed (2014) Retraction notice. J Int Oral Health 6:136

1. Yadav SK, Shrestha S (2014) Rhinosporidiosis of the parotid duct. Case Rep Dent 2014:131794.

- Case Reports In Dentistry (2014) Retracted: Rhinosporidiosis of the parotid duct. Case Rep Dent 2014:382142

1. Calvo-Guirado JL, Gómez-Moreno G, Maté-Sánchez JE, López-Marí L, Delgado-Ruiz R, Romanos GE (2015) New bone formation in bone defects after melatonin and porcine bone grafts: experimental study in rabbits. Clin Oral Implants Res 26:399–406

- No authors listed (2018) Retraction. Clin. Oral Implants Res 29:817

1. Yadav SK, Shetty P (2014) Primary small cell undifferentiated (neuroendocrine) carcinoma of the maxillary sinus. Case Rep Dent 2014:463109

- Case Reports in Dentistry (2014) Retracted: Primary small cell undifferentiated (neuroendocrine) carcinoma of the maxillary sinus. Case Rep Dent 2014:609802

1. Maroli S, Srinath HP, Goinka C, Yadav NS, Bhardwaj A, Varghese RK (2014) Sniffing out pain: An in vivo intranasal study of analgesic efficacy. J Int Oral Health 6:66–71

- No authors listed (2014) Retraction notice. J Int Oral Health 6:111

1. Boquete-Castro A, Gómez-Moreno G, Aguilar-Salvatierra A, Delgado-Ruiz RA, Romanos GE, Calvo-Guirado JL (2015) Influence of the implant design on osseointegration and crestal bone resorption of immediate implants: a histomorphometric study in dogs. Clin Oral Implants Res 26:876–881

- No authors listed (2018) Retraction. Clin Oral Implants Res 29:538

1. Calvo-Guirado JL, Ramírez-Fernández MP, Maté-Sánchez JE, Negri B, Velasquez P, de Aza PN (2015) Enhanced bone regeneration with a novel synthetic bone substitute in combination with a new natural cross-linked collagen membrane: radiographic and histomorphometric study. Clin Oral Implants Res 26:454–464

- No authors listed (2018) Retraction. Clin Oral Implants Res 29:664

1. Saker S, El-Kholany N, El-Gendy A, Fadhil ON, Maria OM (2013) Effect of post space conditioning and luting resin on the retentive strength of fiber-reinforced composite resin posts. J Prosthet Dent (reference not available)

- Saker S, El-Kholany N, El-Gendy A, Fadhil ON, Maria OM (2014) Withdrawn: Effect of post space conditioning and luting resin on the retentive strength of fiber-reinforced composite resin posts. J Prosthet Dent Apr 22. [Epub ahead of print]

1. Gupta H, Puri A, Kumar S (2014) Diagnosis and management of cemental tear: a case report. Gen Dent 62:e12-13

- Gupta H, Pur A, Kumar S (2018) Retraction notice: Diagnosis and management of cemental tear: a case report. Gen Dent. 2014;62(3):e12-e13. Gen Dent 66:8

1. Saker S, El-Kholany N, Sakrana A, Maria OM (2013) Effect of different dentin cleaning techniques on bond strength and the micromorphology of dentin/self-adhesive resin cement interface. J Prosthet Dent (reference not available)

- Saker S, El-Kholany N, Sakrana A, Maria OM (2014) Withdrawn: Effect of different dentin cleaning techniques on bond strength and the micromorphology of dentin/self-adhesive resin cement interface. J Prosthet Dent Apr 29. [Epub ahead of print]

1. Afshar H, Nakhjavani YB, Ahmadi R (2014) The effect of using different rinsing angles on the micro-tensile bond strength of the sealant to the etched enamel. Contemp Clin Dent 5:67–70

- No authors listed (2016) Retraction: The effect of using different rinsing angles on the micro tensile bond strength of the sealant to the etched enamel. Contemp Clin Dent 7:125

1. Jin H, Patil PM, Sharma A (2014) Topical review: the enigma of fibromyalgia. J Oral Facial Pain Headache 28:107–118

- No authors listed (2014) Retraction. J Oral Facial Pain Headache 28:preceding 297

1. Bansal P, Bansal P (2014) Reconstructive surgery with chin block graft and esthetic rehabilitation of missing anterior tooth. J Indian Soc Periodontol 18:263–266

- No authors listed (2014) Notice of retraction. J Indian Soc Periodontol 18:425

1. Mansuri S, Mujeeb A, Hussain SA, Hussain MA (2014) Mandibular third molar impactions in male adults: relationship of operative time and types of impaction on inflammatory complications. J Int Oral Health 6:9–15

- No authors listed (2016) Retraction notice. J Int Oral Health 8:838

1. Ehrlich GD, Hu FZ, Sotereanos N, Sewicke J, Parvizi J, Nara PL, Arciola CR (2014) What role do periodontal pathogens play in osteoarthritis and periprosthetic joint infections of the knee? J Appl Biomater Funct Mater 12:13–20

- No authors listed (2017) Retraction: "What role do periodontal pathogens play in osteoarthritis and periprosthetic joint infections of the knee?" J Appl Biomater Funct Mater 15:e423

1. Passi D, Singh G, Mehta G, Singhal D (2014) Unusually large submandibular epidermoid cyst: A case report, differential diagnosis and therapy. Contemp Clin Dent 5:252–255

- No authors listed (2015) Unusually large submandibular epidermoid cyst: A case report, differential diagnosis and therapy: Retraction. Contemp Clin Dent 6:437

1. Calvo-Guirado JL, Maté-Sánchez JE, Delgado-Ruiz RA, Romanos GE, De Aza-Moya P, Velázquez P (2015) Bone neo-formation and mineral degradation of 4Bone.(®) Part II: histological and histomorphometric analysis in critical size defects in rabbits. Clin Oral Implants Res 26:1402–1406

- No authors listed (2018) Retraction. Clin Oral Implants Res 29:813

1. Sohi R, Gambhir R, Sogi G, Veeresha K, Randhawa A (2014) Dental health status and treatment needs of police personnel of a north Indian state: a cross-sectional study. Ann Med Health Sci Res 4:567–571

- No authors listed (2014) Dental health status and treatment needs of police personnel of a north Indian State: a cross-sectional study: Retraction. Ann Med Health Sci Res 4:S335

1. Feng L, Li H, E LL, Li CJ, Ding Y (2014) Pathological changes in the maxillary sinus mucosae of patients with recurrent odontogenic maxillary sinusitis. Pakistan J Med Sci 30:972–975

- No authors listed (2016) Retraction announcement. Pakistan J Med Sci 32:795

1. Lin F, Yao L, Xiao J, Liu D, Ni Z (2014) MiR-206 functions as a tumor suppressor and directly targets K-Ras in human oral squamous cell carcinoma. Onco Targets Ther 7:1583–1591

- No authors listed (2016) MiR-206 functions as a tumor suppressor and directly targets K-Ras in human oral squamous cell carcinoma [Retraction]. Onco Targets Ther 9:6377

1. He Y, Chen F, Cai Y, Chen S (2015) Knockdown of tumor protein D52-like 2 induces cell growth inhibition and apoptosis in oral squamous cell carcinoma. Cell Biol Int 39:264–271

- No authors listed (2016) Retracted: Knockdown of tumor protein D52-like 2 induces cell growth inhibition and apoptosis in oral squamous cell carcinoma. Cell Biol Int 40:361

1. Nayyar AS, Khan M, Bafna UD, Ahmed S, Chaluvaiah GH (2014) Colposcopy in oral epithelial dysplasia: seeing the unseen, a pilot study. J Cancer Res Ther 10:563–570

- No authors listed (2015) Retraction: Colposcopy in oral epithelial dysplasia: seeing the unseen, a pilot study. J Cancer Res Ther 11:1049

1. Lv J, Zhu YX, Liu YQ, Xue X (2015) Distinctive pathways characterize A. actinomycetemcomitans and P. gingivalis. Mol Biol Rep 42:441–449

- No authors listed (2015) Retraction note to: Distinctive pathways characterize A. actinomycetemcomitans and P. gingivalis. Mol Biol Rep 42:1499

1. Kolaparthy LK, Sanivarapu S, Swarna C, Devulapalli NS (2014) Neutrophil extracellular traps: their role in periodontal disease. J Indian Soc Periodontol 18:693–697

- No authors listed (2015) Neutrophil extracellular traps: their role in periodontal disease: Retraction. J. Indian Soc. Periodontol. 19:128

1. Rao MV, Reddy MV, Sunder SS, Kolasani B, Kiranmai G, Kumar KR (2014) In-dental office screening for diabetes mellitus using gingival crevicular blood. J Int Soc Prev Community Dent 4:S161-165

- Weber-Wulff D (2015) In-dental office screening for diabetes mellitus using gingival crevicular blood: Retraction. J Int Soc Prev Community Dent 5:432

1. de Meireles DA, de Brito TC, Marques AA, Garrido AD, Garcia LF, Sponchiado EC Jr (2015) Micro-computed tomography evaluation of apical transportation and centring ability of Reciproc and WaveOne systems in severely curved root canals. Int Endod J Feb 5. [Epub ahead of print]

- de Meireles DA, de Brito TC, Marques AA, Garrido AD, Garcia LF, Sponchiado EC Jr (2015) Retracted: Micro-computed tomography evaluation of apical transportation and centring ability of Reciproc and WaveOne systems in severely curved root canals. Int Endod J 48:814

1. Calvo-Guirado JL, Delgado Ruiz RA, Ramírez-Fernández MP, Abboud M, Janjic B, Maté Sánchez de Val JE (2016) Histological and histomorphometric analyses of narrow implants, crestal and subcrestally placed in severe alveolar atrophy: a study in foxhound dogs. Clin Oral Implants Res 27:497–504

- No authors listed (2018) Retraction. Clin Oral Implants Res 29:819

1. Nayar S, Bhuminathan S, Mahadevan R (2015) Combination restoration in full mouth rehabilitation. J Pharm Bioallied Sci 7:S288-290

- No authors listed (2016) Retraction: Combination restoration in full mouth rehabilitation. J. Pharm. Bioallied Sci. 8:S192

1. Ni ZY, Lin FO, Liu DF, Xiao J (2015) Decreased microRNA-143 expression and its tumor suppressive function in human oral squamous cell carcinoma. Genet Mol Res 14:6943–6952

- Moura Duarte FA (2016) Retraction of articles with common plagiarism. Genet Mol Res 15:4

1. Maté Sánchez de Val JE, Calvo Guirado JL, Ramírez Fernández MP, Delgado Ruiz RA, Mazón P, De Aza PN (2015) In vivo behavior of hydroxyapatite/β-TCP/collagen scaffold in animal model. Histological, histomorphometrical, radiological, and SEM analysis at 15, 30, and 60 days. Clin Oral Implants Res Aug 7. [Epub ahead of print]

- No authors listed (2018) Retraction. Clin Oral Implants Res 29:816

1. Calvo-Guirado JL, Aguilar-Salvatierra A, Ramírez-Fernández MP, Maté Sánchez de Val JE, Delgado-Ruiz RA, Gómez-Moreno G (2016) Bone response to collagenized xenografts of porcine origin (mp3(®)) and a bovine bone mineral grafting (4BONE(^TM^) XBM) grafts in tibia defects: experimental study in rabbits. Clin Oral Implants Res 27:1039–1046

- No authors listed (2018) Retraction. Clin Oral Implants Res 29:818

1. Atri M, Srivastava D, Kharbanda J, Bugalia A, Yousuf A, Anup N (2015) Occupational stress, salivary cortisol, and periodontal disease: a clinical and laboratory study. J Int Oral Health 7:65–69

- No authors listed (2016) Retraction notice. J Int Oral Health 8:650

1. Madhav VN (2015) Item analysis of multiple-choice questions in teaching prosthodontics. J Dent Educ 79:1314–1319

- No authors listed (2016) Notice of retraction. J Dent Educ 80:1384

1. Saha SG, Vijaywargiya N, Dubey S, Saxena D, Kala S (2015) Evaluation of the incidence of microcracks caused by Mtwo and ProTaper NEXT rotary file systems versus the Self Adjusting File: a scanning electron microscopic study. Int Endod J Nov 24. [Epub ahead of print]

- Saha SG, Vijaywargiya N, Dubey S, Saxena D, Kala S (2016) Retracted: Evaluation of the incidence of microcracks caused by Mtwo and ProTaper NEXT rotary file systems versus the Self Adjusting File: a scanning electron microscopic study. Int Endod J 49:911

1. De Aza PN, Mate-Sanchez de Val JE, Baudin C, Perez Albacete-Martínez C, Armijo Salto A, Calvo-Guirado JL (2016) Bone neoformation of a novel porous resorbable Si-Ca-P-based ceramic with osteoconductive properties: physical and mechanical characterization, histological and histomorphometric study. Clin Oral Implants Res 27:1368–1375

- No authors listed (2018) Retraction. Clin Oral Implants Res 29:537

1. Alvarez C, Benítez A, Rojas L, Pujol M, Carvajal P, Díaz-Zúñiga J, Vernal R (2015) Differential expression of CC chemokines (CCLs) and receptors (CCRs) by human T lymphocytes in response to different Aggregatibacter actinomycetemcomitans serotypes. J Appl Oral Sci 23:580–590

- No authors listed (2016) Retraction. J Appl Oral Sci 24:105

1. Bilgili D, Yilmaz S, Dumani A, Yoldas O (2016) Postoperative pain after irrigation with Vibringe versus a conventional needle: a randomized controlled trial. Int Endod J Feb 29 [Epub ahead of print]

- Bilgili D, Yilmaz S, Dumani A, Yoldas O (2016) Retracted: Postoperative pain after irrigation with Vibringe versus a conventional needle: a randomized controlled trial. Int Endod J 49:813

1. Huang SH, Law CH, Kuo PH, Hu RY, Yang CC, Chung TW, Li JM, Lin LH, Liu YC, Liao EC, Tsai YT, Wei YS, Lin CC, Chang CW, Chou HC, Wang WC, Chang MD, Wang LH, Kung HJ, Chan HL, Lyu PC (2016) MMP-13 is involved in oral cancer cell metastasis. Oncotarget 7:17144–17161

- Huang SH, Law CH, Kuo PH, Hu RY, Yang CC, Chung TW, Li JM, Lin LH, Liu YC, Liao EC, Tsai YT, Wei YS, Lin CC, Chang CW, Chou HC, Wang WC, Chang MD, Wang LH, Kung HJ, Chan HL, Lyu PC (2016) Retraction: MMP-13 is involved in oral cancer cell metastasis. Oncotarget 7:48851

1. Cintra LT, Benetti F, Ferreira LL, Rahal V, Ervolino E, Jacinto Rde C, Gomes Filho JE, Briso AL (2016) Evaluation of an experimental rat model for comparative studies of bleaching agents. J Appl Oral Sci 24:171–180

- J Appl Oral Sci (2016) Retraction. J Appl Oral Sci 24:308

1. Sahana S, Vasa AA, Geddam D, Reddy VK, Nalluri S, Velagapudi N (2016) Effectiveness of chemomechanical caries removal agents Papacarie(®) and Carie-Care^TM^ in primary molars: An in vitro study. J Int Soc Prev Community Dent 6:S17-22

- No authors listed (2016) Retraction: Effectiveness of chemomechanical caries removal agents Papacarie(®) and Carie-Care^TM^ in primary molars: An in vitro study. J Int Soc Prev Community Dent 6:391

1. Calvo-Guirado JL, Maté-Sánchez de Val JE, Delgado-Ruiz RA, Fernández Domínguez M, Orlato Rossetti PH, Gehrke SA (2016) A new cervical implant design compared with standard design in order to increase peri-implant hard and soft tissue behavior: histomorphometric and histological study in dogs. Clin Oral Implants Res Sep 5 [Epub ahead of print]

- No authors listed (2018) Retracted: A new cervical implant design compared with standard design in order to increase peri‐implant hard and soft tissue behavior: histomorphometric and histological study in dogs. Clin Oral Implants Res 29:815

1. Lin F, Yao L, Bhikoo C, Guo J (2016) Impact of fixed orthodontic appliance or clear-aligner on daily performance, in adult patients with moderate need for treatment. Patient Prefer Adherence 10:1639–1645

- No authors listed (2016) Impacts of fixed orthodontic appliance and clear-aligner on daily performance in adult patients with moderate need for treatment [Retraction]. Patient Prefer Adherence 10:2321

1. Acharya S, Mandal PK (2016) Salivary IgA and dental caries in HIV patients: A pilot study. J Indian Soc Pedod Prev Dent 34:341–347

- No authors listed (2017) Retraction: Salivary IgA and dental caries in HIV patients: A pilot study. J Indian Soc Pedod Prev Dent 35:98

1. Passi D, Singh G, Dutta S, Sharma S, Mishra S, Gupta C (2014) Honey extract as medicament for treatment of dry socket: an ancient remedy rediscovered-case series and literature review. J Maxillofac Oral Surg Nov 21 [Epub ahead of print]

- Passi D, Singh G, Dutta S, Sharma S, Mishra S, Gupta C (2016) Retracted article: Honey extract as medicament for treatment of dry socket: an ancient remedy rediscovered-case series and literature review. J Maxillofac Oral Surg 15:345

1. Velosa C, Shi Q, Stevens TM, Chiosea SI, Purgina B, Carroll W, Rosenthal E, Morlandt A, Loree T, Brandwein-Weber MS (2017) Worst pattern of invasion and occult cervical metastases for oral squamous carcinoma. Head Neck Mar 28. [Epub ahead of print]

- Velosa C, Shi Q, Stevens TM, et al (2017) Retracted: Worst pattern of invasion and occult cervical metastases for oral squamous carcinoma. Head Neck 39:2142

1. Gunawardane S, Kapugama K (2016) A rare pathological entity of multiple calcified hyperplastic dental follicles. Case Rep Dent 2016:4190827

- Case Reports in Dentistry (2017) Retracted: A rare pathological entity of multiple calcified hyperplastic dental follicles. Case Rep Dent 2017:5149065

1. Fee L (2017) Socket preservation. Br Dent J 222:579–582

- Fee L (2017) Retraction note: Socket preservation. Br Dent J 223:741

1. Hwang JTK, Gu YR, Dickson BJ, Shen M, Ralhan R, Walfish PG, Mock D, Pritzker KPH (2017) Straticyte demonstrates prognostic value over oral epithelial dysplasia grade for oral potentially malignant lesion assessment. Oral Oncol 72:1–6

- Hwang JTK, Gu YR, Dickson BJ, Shen M, Ralhan R, Walfish PG, Mock D, Pritzker KPH (2018) Retracted: Straticyte demonstrates prognostic value over oral epithelial dysplasia grade for oral potentially malignant lesion assessment. Oral Oncol 77:138

1. Shah AF, Batra M, Ishrat A (2016) Transition in dental treatment utilization in Jammu And Kashmir, India - A 10 year retrospective study. Nepal J Epidemiol 6:631–639

- Shah AF, Batra M, Ishrat A (2017) Retraction notice: Transition in dental treatment utilization in Jammu And Kashmir, India - A 10 year retrospective study. Nepal J Epidemiol 7:699

1. Yang HW, Tang XS, Tian ZW, Wang Y, Yang WY, Hu JZ (2017) Effects of nano-hydroxyapatite/ polyetheretherketone-coated, sandblasted, large-grit, and acid-etched implants on inflammatory cytokines and osseointegration in a peri-implantitis model in beagle dogs. Med Sci Monit 23:4601–4611

- Yang HW, Tang XS, Tian ZW, Wang Y, Yang WY, Hu JZ (2018) Retracted: Effects of nano-hydroxyapatite/ polyetheretherketone-coated, sandblasted, large-grit, and acid-etched implants on inflammatory cytokines and osseointegration in a peri-implantitis model in beagle dogs. Med Sci Monit 24:8803

1. Lin X, Zhou N, Huang X, Song S, Li H (2018) Anterior maxillary segmental distraction osteogenesis for treatment of maxillary hypoplasia in patients with repaired cleft palate. J Craniofac Surg 29:e480–484

- No authors listed (2018) Anterior maxillary segmental distraction osteogenesis for treatment of maxillary hypoplasia in patients with repaired cleft palate: Retraction. J Craniofac Surg Nov 13.

1. Rizzo R, Quaranta A, De Paoli M, Rappelli G, Piemontese M (2018) Three-dimensional bone augmentation and immediate implant placement via transcrestal sinus lift: 8-year clinical outcomes. Int J Periodontics Restorative Dent 38:423–429

- No authors listed (2018) Notice of retraction. Int J Periodontics Restorative Dent 38:516

1. Alsulaimani RS (2018) Immediate and delayed repair of 2 sizes of furcal perforations in dogs’ teeth using mineral trioxide aggregate cement. J Endod 44:1000–1006

- No authors listed (2018) Retraction notice. J Endod 44:1888

1. Lan R, Hadj-Saïd M, Foletti JM, Massereau E, Chossegros C (2018) Osteonecrosis of the jaw in patients traited by denosumab for malignant bone disease: descriptive study on 9 cases. Med Oral Patol Oral Cir Bucal Apr 22. [Epub ahead of print]

- Lan R, Hadj-Saïd M, Foletti JM, Massereau E, Chossegros C (2018) Withdrawn: Osteonecrosis of the jaw in patients traited by denosumab for malignant bone disease: descriptive study on 9 cases. Med Oral Patol Oral Cir Bucal (reference not available)

1. Moshonov J, Peretz B, Brown T, Rotstein I (2004) Cleaning of the root canal using Nd:YAP laser and its effect on the mineral content of the dentin. J Clin Laser Med Surg 22:87–89

- No authors listed (2015) Retraction of: J Clin Las Med Surg 2004;22(2):87-89 DOI: 10.1089/104454704774076127. Photomed Laser Surg 33:576

1. Various authors (2004) Abstracts from international literature. Int J Oral Maxillofac Surg 33:315-319

- No authors listed (2011) Withdrawn: Abstracts from international literature. Int J Oral Maxillofac Surg Jan 20. [Epub ahead of print]

1. Perrott DH (2004) Book review. Int J Oral Maxillofac Surg 33:320

- Perrott DH (2011) Withdrawn: Book review. Int J Oral Maxillofac Surg Jan 20. [Epub ahead of print]

1. Kos M (2011) Bisphosphonates promote jaw osteonecrosis through facilitating bacterial colonisation. Med Hypotheses 77:214–215

- No authors listed (2012) Retraction. Bisphosphonates promote jaw osteonecrosis through facilitating bacterial colonisation. [Med. Hypotheses 77 (2011) 214-215]. Med Hypotheses 78:352

1. Hiremath VP, Rao CB, Naiak V, Prasad KV (2013) Anti-inflammatory effect of vitamin D on gingivitis: a dose response randomised controlled trial. Indian J Public Health 57:29–32

- No authors listed (2013) Retraction. Indian J Public Health 57:77

1. Schwendicke F (2016) Modern concepts for caries tissue removal. J Esthet Restor Dent Feb 15. [Epub ahead of print]

- No authors listed (2016) Retraction statement: Modern concepts for caries tissue removal. J Esthet Restor Dent 28:136

1. Khung R, Suansuwan NS (2013) Effect of gold sputtering on the adhesion of porcelain to cast and machined titanium. J Prosthet Dent 110:101–106

- Khung R, Suansuwan NS (2013) Withdrawn. Duplicate: Effect of gold sputtering on the adhesion of porcelain to cast and machined titanium. J Prosthet Dent (no reference available)

1. Velleuer E, Dietrich R, Frohnmayer A, Pomjanski N, Hays LE, Biersterfeld S (2017) Prevalence and clinical significance of visible oral lesions in patients with Fanconi anemia at risk for head and neck cancer. Curr Drug Targets May 24. [Epub ahead of print]

- No authors listed (2017) Withdrawn: Prevalence and clinical significance of visible oral lesions in patients with Fanconi anemia at risk for head and neck cancer. Curr Drug Targets (no reference available)
